# Nutritional Strategy for Cancer—From Prevention to Aftercare

**DOI:** 10.3390/nu16101437

**Published:** 2024-05-10

**Authors:** Jakub Klekowski, Mariusz Chabowski

**Affiliations:** 1 Department of Nursing and Obstetrics, Division of Anesthesiological and Surgical Nursing, Faculty of Health Science, Wroclaw Medical University, 50-367 Wroclaw, Poland; klekowski.jakub@gmail.com; 2 Department of Surgery, 4th Military Clinical Hospital, 50-981 Wroclaw, Poland; 3 Department of Clinical Surgical Sciences, Faculty of Medicine, Wroclaw University of Science and Technology, 50-556 Wroclaw, Poland

In recent decades, there has been a noteworthy increase in the efficacy of oncological treatments for a variety of neoplasms, which has improved the overall results and survival rates in cancer therapy. Even if pharmaceutical treatment is evolving quickly, supportive care is still necessary and, when paired with surgery, radiotherapy, and chemotherapy, could work as an adjuvant to guarantee improved outcomes. A growing number of articles (roughly 57 thousand) about nutrition in cancer have been published in the last ten years, according to PubMed. It is challenging to keep oneself up to date with the latest developments in this discipline due to the vast amount of new research that is being conducted. The Special Issue ‘Nutritional Strategy for Cancer—from Prevention to Aftercare’ aimed at collecting research with interesting findings and reviews discussing the latest discoveries.

1. Characteristics and conclusions of the featured studies.

Lung cancer is the most common malignancy in the world, and has a high lethality; therefore, it generates a great burden for healthcare systems in the world [1]. Polański et al. reviewed the role of a diet as a supportive factor in lung cancer treatment and reported some interesting findings [2]. The authors point out that polyunsaturated fatty acids (PUFAs), particularly ω-3 PUFA, which includes eicosapentaenoic acid (EPA), may lessen inflammation, reduce muscle loss, and alter important pathways that drive the development of lung cancer [3,4]. The importance of electrolyte balance was covered in the study. With an emphasis on the potential advantages of vitamins A and C, the authors provide some information on the functions of antioxidants in the prevention and treatment of lung cancer. Dietary interventions were also explored. Although it was noted that the combination of radiation, chemotherapy, and a ketogenic diet enhanced the steady-state levels of proteins damaged by oxidative stress, the ketogenic diet did not appear to offer any significant advantages in terms of overall results. It was also discovered that, among smokers who were genetically predisposed to cigarettes’ harmful effect, diet had a negligible impact. Interestingly, some research on PUFA consumption was assessed. Patients who received EPA supplements consumed more protein and energy, gained more body mass, improved their appetite, felt less fatigued, and had better cognitive abilities. Patients receiving chemotherapy showed improved oxidative and inflammatory markers when EPA and DHA were added to their diets. According to certain statistics, the aforementioned intervention may enhance overall survival and lower surgical complications [5,6]. According to the review authors, the goal of any planned intervention should be to prevent malnutrition. Future research is challenged by the paucity of data on effective diet-related preventive strategies for lung cancer, which hinders the ability to draw valid conclusions [2].

Theinel et al. examined the role of ω-3 PUFA in breast cancer prevention, and how it complements traditional therapy [7]. PUFA influence was examined in both animal and human models in this thorough analysis [7]. The study found that supplementing with ω-3 PUFA, when combined with radiation and chemotherapy for patients with breast cancer, can lower pain symptoms, prevent cachexia–anorexia syndrome, increase weight in cancer patients, slow down the growth and division of cancer cells, lessen inflammation, enhance the effects of chemotherapy, and increase the overall survival rate of cancer patients [8]. Regarding prevention, it has been observed that women who consume higher amounts of total ω-3 PUFAs than ω-6 PUFAs are at a lower risk of breast cancer [9]. The importance of ω-3 PUFA supplementation, as a supplemental treatment in addition to chemotherapy or other traditional anticancer medicines, is highlighted by the authors′ conclusion. Compared to employing therapies or ω-3 PUFAs independently, the researchers observe that its noteworthy efficacy when combination with these treatments results in reduced tumor growth. However, preclinical studies leave us unsure of the optimal ω-3 PUFA dosage for pharmaconutrition, anticancer effects, or breast cancer prevention [7].

The state of the art for food intended for special medical purposes (FSMP) for cancer patients was examined critically by Frydrych et al. [10] Zn, Cu, Se, Fe, and Mn, five necessary elements, were assessed in relation to FSMP. Although the evaluation is deep, certain bullet points stand out. Zinc influences immune system activity and controls inflammatory response. Zinc homeostasis disturbances are observed in cancer patients, which means that careful monitoring of the right amounts of this essential component is required during nutritional therapy. The levels of copper are correlated with cancer stage; therefore, it is critical to validate the supply and bioavailability of copper from FSMP in order to reduce the possibility of distorting test results related to cancer recurrence. Cancer cells often alter their iron metabolism to promote iron accumulation. This allows for increased iron uptake and storage, as well as decreased iron exportation, either separately or simultaneously. Considering the significant changes in metabolism that are seen in cancer, special attention needs to be paid to investigating the relationship between iron metabolism and the nutrients provided by FSMP. It is noteworthy that there are no clear-cut technical guidelines for manufacturers in terms of the elemental makeup of FSMPs meant for cancer patients. In addition, there is a worrying pattern that producers seem to ignore or fail to comply with the scientific literature that outlines the necessary specifications of FSMPs for cancer treatment. The seeming inability of producers to determine whether ingredients present in excess or insufficient quantities would expose cancer patients to additional carcinogenic hazards is significant. As a result, it is imperative that FSMPs have unique technical and quality criteria that are suited to the needs of cancer patients, placing them within the same regulatory and supervisory framework as pharmaceutical goods [10].

Research has illustrated the potential utility of natural polyphenols in both the prophylaxis and therapeutic intervention of cancer. These compounds are believed to exert their effects through diverse mechanisms, encompassing antioxidant properties, anti-inflammatory actions, and modulation of various molecular pathways implicated in carcinogenesis [11]. Markowska et al. conducted a review focusing on the anticancer properties of fisetin, a representative flavonol. Their analysis suggests that fisetin exhibits anti-cancer efficacy in female malignancies [12]. Its capacity to upregulate pro-apoptotic genes, such as caspase 3/8 and Bax, while downregulating the expression of the anti-apoptotic protein Bcl-2, is thought to be responsible for the anti-cancer effect in general. Fisetin has been linked to the promotion of necroptosis and inhibition of the Wnt/β-catenin pathway, in addition to important signaling cascades, such as PI3K/AKT/mTOR, MAPK, and ERK1/2. It is also recognized for its suppression of cyclin-dependent cell cycle activities and downregulation of nuclear factor NF-κB [13,14,15,16,17,18]. The research, according to the authors, suggests that several flavonoids, including fisetin, may have therapeutic value in the fight against cervical cancer. An important role for the arachidonic acid signaling pathway appears in the carcinogenic processes connected to this kind of cancer. Meanwhile, flavonoids have the ability to block this pathway, indicating their potential use in chemopreventive measures, as well as anticancer treatments [19]. Among the array of studies pertaining to cervical cancer, one particularly intriguing finding highlighted a synergistic effect observed between fisetin and sorafenib, wherein their combined administration notably augmented apoptosis [20]. Within the context of breast cancer (BC), there exists observed epigenetic regulation of the human epidermal growth factor receptor 2 (HER-2). Notably, fisetin has demonstrated its effectiveness in BC cell lines characterized by overexpression of the HER-2/neu receptor. Furthermore, almost 70% of BC cases have the PI3K/AKT/mTOR signaling pathway activated, which is linked to unfavorable clinical characteristics and a poor prognosis. It has also been discovered that fisetin is able to target and inhibit this signaling pathway. Studies have demonstrated that fisetin possesses the ability to diminish the proliferation and invasiveness of cancer cells, attenuate their metastatic potential and, in animal models, impede the growth of cancerous tumors [21]. Utilizing nanotechnology holds promise for enhancing the bioavailability of fisetin, thereby potentially amplifying its clinical efficacy. The development and application of fisetin delivery nanosystems have predominantly focused on breast cancer, suggesting a targeted approach in the utilization of this technology [22]. The reviewers conclude that while fisetin shows promise in various roles, such as chemopreventive management, adjuvant therapy, and synergistic combination with conventional cancer drugs, its potential requires further validation. This includes comprehensive assessment of long-term effects, balancing therapeutic benefits against potential adverse outcomes, and determining optimal dosage regimens. Additionally, investigating combination strategies, particularly those involving metabolic inhibitors, holds the potential to optimize treatment modalities or circumvent the mechanisms of drug resistance. Notably, two clinical trials are presently in the planning stages to address these aspects [12].

Grant focused on the careful evaluation of the reliability of earlier research regarding the role of diet in cancer risk and presented a compelling study. The case–control and prospective cohort studies were examined by the author, who concentrated on the relationships between the incidence of bladder, breast, colorectal, and gastric cancer, and the consumption of red and processed meat. These results were compared to a recent prospective cohort study that looked into the connection between different food groups and the chance of developing chronic diseases. Notably, for a thorough analysis, nutritional consumption was assessed in this study every four years. Grant concluded that the assessment of food consumption ought to take place no more than four years prior to diagnosis, preferably sooner. Even while assessments made at early stages may show a lower chance of uncovering meaningful connections, they should still be taken into consideration if the data are available. Furthermore, when feasible, the preference should lie with case–control or nested case–control studies over cohort studies, as they streamline data collection efforts and conserve biological specimens. Moreover, when evaluating the effects of diet on disease risk, observational studies with shorter follow-up periods or tighter time intervals between disease diagnosis and dietary data collection should be given equivalent or enhanced importance in comparison to those with longer time intervals. Eventually, a daring assertion was made, suggesting the imperative revision of previous meta-analyses encompassing both case–control and cohort studies examining the nexus between dietary intake and disease outcomes. According to the author, this revision should include meticulous adjustments for the duration or interval of follow-up periods whenever feasible, ensuring the accuracy and reliability of the findings [23].

2. Implications and possible directions.

The studies highlighted several notable findings. The potential of dietary interventions, possibly complemented by FSMPs, warrants consideration in the daily care of cancer patients. Future studies in nutrition should take into account the conclusions drawn by Grant.

Among the plethora of compelling data presented, evidence regarding PUFAs, particularly omega-3 PUFAs, is noteworthy. The incorporation of EPA and DHA into nutritional regimes as adjuncts to treatment is supported by the research and offers benefits to patients. However, future research efforts should focus on enriching lipidomic analysis. Specifically, exploring the landscape of PUFAs within cancerous tissues and blood samples from patients supplementing their diet with omega-3 and receiving other nutritional supplements holds promise for further insights.

## References

[1] Siegel R.L., Miller K.D., Fedewa S.A., Ahnen D.J., Meester R.G.S., Barzi A., Jemal A. (2017). Colorectal cancer statistics, 2017. CA Cancer J. Clin..

[2] Polański J., Świątoniowska-Lonc N., Kołaczyńska S., Chabowski M. (2023). Diet as a Factor Supporting Lung Cancer Treatment—A Systematic Review. Nutrients.

[3] Arends J., Bachmann P., Baracos V., Barthelemy N., Bertz H., Bozzetti F., Fearon K., Hütterer E., Isenring E., Kaasa S. (2017). ESPEN guidelines on nutrition in cancer patients. Clin. Nutr..

[4] Pappalardo G., Almeida A., Ravasco P. (2015). Eicosapentaenoic acid in cancer improves body composition and modulates metabolism. Nutrition.

[5] Finocchiaro C., Segre O., Fadda M., Monge T., Scigliano M., Schena M., Tinivella M., Tiozzo E., Catalano M.G., Pugliese M. (2012). Effect of *n*-3 fatty acids on patients with advanced lung cancer: A double-blind, placebo-controlled study. Br. J. Nutr..

[6] Sánchez-Lara K., Turcott J.G., Juárez-Hernández E., Nuñez-Valencia C., Villanueva G., Guevara P., De la Torre-Vallejo M., Mohar A., Arrieta O. (2014). Effects of an oral nutritional supplement containing eicosapentaenoic acid on nutritional and clinical outcomes in patients with advanced non-small cell lung cancer: Randomised trial. Clin. Nutr..

[7] Theinel M.H., Nucci M.P., Alves A.H., Dias O.F.M., Mamani J.B., Garrigós M.M., Oliveira F.A., Rego G.N.A., Valle N.M.E., Cianciarullo G. (2023). The Effects of Omega-3 Polyunsaturated Fatty Acids on Breast Cancer as a Preventive Measure or as an Adjunct to Conventional Treatments. Nutrients.

[8] Samanta C., Tewari S., Chakraborty D., Vaishnav S. (2022). Omega-3 Fatty Acid and its Protective Effect against Cancer and Cancer-related Complication. J. Pharm. Res. Int..

[9] Fabian C.J., Kimler B.F., Hursting S.D. (2015). Omega-3 fatty acids for breast cancer prevention and survivorship. Breast Cancer Res..

[10] Frydrych A., Krośniak M., Jurowski K. (2023). The Role of Chosen Essential Elements (Zn, Cu, Se, Fe, Mn) in Food for Special Medical Purposes (FSMPs) Dedicated to Oncology Patients—Critical Review: State-of-the-Art. Nutrients.

[11] Zhou Y., Zheng J., Li Y., Xu D.-P., Li S., Chen Y.-M., Li H.-B. (2016). Natural Polyphenols for Prevention and Treatment of Cancer. Nutrients.

[12] Markowska A., Antoszczak M., Kacprzak K., Markowska J., Huczyński A. (2023). Role of Fisetin in Selected Malignant Neoplasms in Women. Nutrients.

[13] Syed D.N., Afaq F., Maddodi N., Johnson J.J., Sarfaraz S., Ahmad A., Setaluri V., Mukhtar H. (2011). Inhibition of human melanoma cell growth by the dietary flavonoid fisetin is associated with disruption of Wnt/β-catenin signaling and decreased mitf levels. J. Investig. Dermatol..

[14] Suh Y., Afaq F., Johnson J.J., Mukhtar H. (2009). A plant flavonoid fisetin induces apoptosis in colon cancer cells by inhibition of COX2 and Wnt/EGFR/NF-κB-signaling pathways. Carcinogenesis.

[15] Carmi Y.K., Mahmoud H., Khamaisi H., Adawi R., Gopas J., Mahajna J. (2020). Flavonoids Restore Platinum Drug Sensitivity to Ovarian Carcinoma Cells in a Phospho-ERK1/2-Dependent Fashion. Int. J. Mol. Sci..

[16] Jafarzadeh S., Baharara J., Tehranipour M. (2021). Apoptosis Induction with Combined Use of Cisplatin and Fisetin in Cisplatin- Resistant Ovarian Cancer Cells (A2780). Avicenna J. Med. Biotechnol..

[17] Liu Y., Cao H., Zhao Y., Shan L., Lan S. (2022). Fisetin-induced cell death in human ovarian cancer cell lines via zbp1-mediated necroptosis. J. Ovarian Res..

[18] Kubina R., Krzykawski K., Kabała-Dzik A., Wojtyczka R.D., Chodurek E., Dziedzic A. (2022). Fisetin, a Potent Anticancer Flavonol Exhibiting Cytotoxic Activity against Neoplastic Malignant Cells and Cancerous Conditions: A Scoping, Comprehensive Review. Nutrients.

[19] Yarla N.S., Bishayee A., Sethi G., Reddanna P., Kalle A.M., Dhananjaya B.L., Dowluru K.S., Chintala R., Duddukuri G.R. (2016). Targeting arachidonic acid pathway by natural products for cancer prevention and therapy. Semin. Cancer Biol..

[20] Lin M.-T., Lin C.-L., Lin T.-Y., Cheng C.-W., Yang S.-F., Lin C.-L., Wu C.-C., Hsieh Y.-H., Tsai J.-P. (2016). Synergistic effect of fisetin combined with sorafenib in human cervical cancer HeLa cells through activation of death receptor-5 mediated caspase-8/caspase-3 and the mitochondria-dependent apoptotic pathway. Tumor Biol..

[21] Sun X., Ma X., Li Q., Yang Y., Xu X., Sun J., Yu M., Cao K., Yang L., Yang G. (2018). Anti-cancer effects of fisetin on mammary carcinoma cells via regulation of the PI3K/Akt/mTOR pathway: In vitro and in vivo studies. Int. J. Mol. Med..

[22] Feng C., Yuan X., Chu K., Zhang H., Ji W., Rui M. (2019). Preparation and optimization of poly (lactic acid) nanoparticles loaded with fisetin to improve anti-cancer therapy. Int. J. Biol. Macromol..

[23] Grant W.B. (2023). Long Follow-Up Times Weaken Observational Diet–Cancer Study Outcomes: Evidence from Studies of Meat and Cancer Risk. Nutrients.

